# Factors Associated With Liver Transplant Referral Among Patients With Cirrhosis at Multiple Safety-Net Hospitals

**DOI:** 10.1001/jamanetworkopen.2023.17549

**Published:** 2023-06-08

**Authors:** Mignote Yilma, Nicole J. Kim, Amy M. Shui, Michele Tana, Charles Landis, Ariana Chen, Saroja Bangaru, Neil Mehta, Kali Zhou

**Affiliations:** 1 Department of General Surgery, University of California, San Francisco; 2 National Clinician Scholars Program, University of California, San Francisco; 3 Division of Gastroenterology, University of Washington, Seattle; 4 Department of Epidemiology and Biostatistics, University of California, San Francisco; 5 Department of Gastroenterology and Hepatology, Zuckerberg San Francisco General Hospital, San Francisco, California; 6 Department of Medicine, University of Michigan, Ann Arbor; 7 Division of Gastrointestinal and Liver Diseases, University of Southern California, Los Angeles; 8 Department of Gastroenterology, University of California, San Francisco

## Abstract

**Importance:**

A high proportion of underserved patients with cirrhosis receive care at safety-net hospitals (SNHs). While liver transplant (LT) can be a life-saving treatment for cirrhosis, data on referral patterns from SNHs to LT centers are lacking.

**Objective:**

To identify factors associated with LT referral within the SNH context.

**Design, Setting, and Participants:**

This retrospective cohort study included 521 adult patients with cirrhosis and model for end-stage liver disease–sodium (MELD-Na) scores of 15 or greater. Participants received outpatient hepatology care at 3 SNHs between January 1, 2016, and December 31, 2017, with end of follow-up on May 1, 2022.

**Exposures:**

Patient demographic characteristics, socioeconomic status, and liver disease factors.

**Main Outcomes and Measures:**

Primary outcome was referral for LT. Descriptive statistics were used to describe patient characteristics. Multivariable logistic regression was performed to evaluate factors associated with LT referral. Multiple chained imputation was used to address missing values.

**Results:**

Of 521 patients, 365 (70.1%) were men, the median age was 60 (IQR, 52-66) years, most (311 [59.7%]) were Hispanic or Latinx, 338 (64.9%) had Medicaid insurance, and 427 (82.0%) had a history of alcohol use (127 [24.4%] current vs 300 [57.6%] prior). The most common liver disease etiology was alcohol associated liver disease (280 [53.7%]), followed by hepatitis C virus infection (141 [27.1%]). Median MELD-Na score was 19 (IQR, 16-22). One hundred forty-five patients (27.8%) were referred for LT. Of these, 51 (35.2%) were wait-listed, and 28 (19.3%) underwent LT. In a multivariable model, male sex (adjusted odds ratio [AOR], 0.50 [95% CI, 0.31-0.81]), Black race vs Hispanic or Latinx ethnicity (AOR, 0.19 [95% CI, 0.04-0.89]), uninsured status (AOR, 0.40 [95% CI, 0.18-0.89]), and hospital site (AOR, 0.40 [95% CI, 0.18-0.87]) were associated with lower odds of being referred. Reasons for not being referred (n = 376) included active alcohol use and/or limited sobriety (123 [32.7%]), insurance issues (80 [21.3%]), lack of social support (15 [4.0%]), undocumented status (7 [1.9%]), and unstable housing (6 [1.6%]).

**Conclusions:**

In this cohort study of SNHs, less than one-third of patients with cirrhosis and MELD-Na scores of 15 or greater were referred for LT. The identified sociodemographic factors negatively associated with LT referral highlight potential intervention targets and opportunities to standardize LT referral practices to increase access to life-saving transplant among underserved patients.

## Key Points

**Question:**

What are the factors associated with liver transplant referral for patients with cirrhosis and a model for end-stage liver disease–sodium score of 15 or greater in safety-net hospitals?

**Findings:**

In this cohort study of 521 patients in safety-net hospitals, 145 (28%) were referred; of these, 51 (35%) were wait-listed and 28 (19%) underwent liver transplant. Factors associated with lower odds of referral included male sex, Black race, uninsured status, and hospital site.

**Meaning:**

These findings suggest that the first barrier to liver transplant for underserved patients with cirrhosis is at the referral stage, and interventions targeting this step are needed.

## Teaser

This cohort study evaluates the factors associated with liver transplant referral among adult outpatients with end-stage liver disease and an indication for transplant at safety-net hospitals.

## Introduction

End-stage liver disease (ESLD) due to cirrhosis and its associated complications is a leading cause of death among US adults,^1^ with the highest prevalence among underserved populations who primarily access care at safety-net hospitals (SNHs).^2,3^ At SNHs, essential care is provided to vulnerable patients regardless of their ability to pay.^4^ Approximately 33% of cirrhosis-related hospitalizations occur in SNHs.^5^ This is partly driven by the high prevalence of cirrhosis among members of racial and ethnic minority groups,^6^ who are 26% to 54% more likely to be hospitalized in SNHs than non-SNHs,^5^ and the high prevalence of cirrhosis among low-income patients (<$20 000/y).^3,5^

Liver transplant (LT) is the only life-saving procedure available for patients with ESLD. To receive LT, patients must be referred, evaluated, and registered on the United Network for Organ Sharing (UNOS) wait list. While UNOS collects detailed information on wait-listed patients, there are limited data on prelisting LT referral patterns and outcomes. Importantly, no prior study, to our knowledge, has evaluated the LT referral continuum among patients who receive care at SNHs. Black and Hispanic patients with cirrhosis have higher odds of being hospitalized at SNHs, with Black patients having higher odds of in-hospital mortality compared with White patients.^5,7,8^ A previous study assessing LT within the SNH context^9^ used the proportion of patients with Medicaid or no insurance to characterize a hospital as an SNH; within this context, the investigators found 8.0% of patients (n = 885) received LT at a high-burden SNH. A more recent study of 161 patients with hepatocellular carcinoma in SNHs^10^ found that only one-third of the patients were referred for LT evaluation, only 5% were wait-listed, and 1.3% received a transplant.

Patients with cirrhosis treated at SNHs have high mortality.^5^ Most current studies^11,12,13^ focus on identifying factors associated with LT once a patient is referred for evaluation, rather than referral rates among the eligible population with ESLD, where significant drop-offs are anticipated. The purpose of this multicenter cohort study was to evaluate the frequency of and factors associated with LT referral for adult outpatients with ESLD and an indication for LT (model for end-stage liver disease–sodium [MELD-Na] score ≥15) receiving care at SNHs. Given the disproportionate burden of ESLD among individuals from racial and ethnic minority groups, uninsured patients, and patients with Medicaid coverage, assessing the LT referral landscape within SNHs provides a unique opportunity to evaluate barriers to LT referral among the most vulnerable patients.

## Methods

### Data Source

This retrospective cohort study included patients from 3 large urban SNH systems: Zuckerberg San Francisco General Hospital (ZSFGH; San Francisco, California), Harborview Medical Center (HMC; Seattle, Washington), and Los Angeles County plus University of Southern California Medical Center (LAC plus USC; Los Angeles). The study followed the Strengthening the Reporting of Observational Studies in Epidemiology (STROBE) reporting guideline and was approved by the University of California, San Francisco (UCSF), University of Washington, USC, and UCLA (University of California, Los Angeles) institutional review boards. Patients did not provide informed consent because the study was entirely retrospective with minimal risk to privacy.

As part of the San Francisco Health Network, ZSFGH provides care to over 100 000 patients in San Francisco annually. Harborview Medical Center is a comprehensive health care facility that provides specialized care for the most vulnerable residents of King County and the Pacific Northwest region with over 260 000 annual clinic visits. Los Angeles County plus USC Medical Center is under the Los Angeles County Department of Public Health and consists of 26 health centers and 4 acute care hospitals, providing care to over 750 000 unique patients yearly. All hospital systems provide care regardless of an individuals’ ability to pay or immigration status.

The hepatology clinic at ZSFGH is staffed by nontransplant hepatologists; the primary transplant center for referrals is UCSF. The hepatology clinic at HMC is staffed by both nontransplant and transplant hepatologists, with transplant hepatologists also rotating at the University of Washington transplant center, the primary transplant center for HMC referrals. Hepatology clinics at LAC plus USC are staffed by both nontransplant and transplant hepatologists, with transplant hepatologists also rotating at Keck Hospital of USC transplant center. Referrals from LAC plus USC are sent to either Keck Hospital of USC or UCLA, depending on patient insurance. The distance between the SNHs and their corresponding transplant centers ranged from 1 to 17 miles.

At all 3 SNHs, physicians initiate patient referrals with documents being faxed or electronically sent through the medical record system to the local transplant center contracted with the patients’ insurance. The transplant center then reaches out to the patient to schedule an evaluation visit. Baseline patient characteristics and referral data were obtained from the SNH sites, while LT evaluation and outcome data were obtained from transplant centers.

### Study Population

We included adult patients (aged ≥18 years) with an *International Statistical Classification of Diseases and Related Health Problems, Tenth Revision*, code for cirrhosis (K70.30, K70.31, K71.7, K74.3, K74.4, K74.5, K74.60, or K74.69), at least 1 outpatient hepatology clinic visit, and a MELD-Na score of 15 or greater between January 1, 2016, and December 31, 2017. The MELD-Na score cutoff was selected as this is the recommended threshold for consideration of LT.^14,15^ Study entry was the date of index liver clinic visit with the MELD-Na score of 15 or greater. Patients were then followed up until December 31, 2019, to assess for the primary outcome of LT referral (referred vs not referred). For patients who were referred, postreferral outcomes such as evaluation, wait-listing, and mortality were ascertained until May 1, 2022. We excluded patients referred for LT prior to the start of the study period.

### Outcomes and Exposures

Variables included demographic, socioeconomic, and disease-related variables. Demographic variables include age, sex, race and ethnicity, marital status (single, married, or divorced, separated, or widowed), birth status outside the US (yes or no), documentation status (documented [permanent resident, green card or visa holder, or citizen], undocumented, or unknown or missing), and primary language (English, Spanish, or other). Given the differences in waitlisting and transplant outcomes by race and ethnicity, we wanted to assess whether a patient’s race and ethnicity impacted their referral outcome. That information, as captured by electronic health record, was categorized into 4 mutually exclusive groups (Hispanic or Latinx, non-Hispanic Black, non-Hispanic White, and non-Hispanic other). Other race and ethnicity included American Indian or Alaska Native, Asian American or Pacific Islander, and more than 1 race. Hispanic or Latinx patients were used as the reference group in this analysis as they represented the largest proportion of the cohort. Socioeconomic status variables included housing stability (stable, unstable, or unknown or missing) and insurance type (private, Medicaid, Medicare, or uninsured). Unstable housing was defined as single room occupation, homeless, or being housed in a shelter. Disease-related variables included liver disease etiology (alcohol-associated liver disease [ALD], nonalcoholic steatohepatitis, chronic hepatitis B virus, chronic hepatitis C virus, and other), diabetes (yes or no), hypertension (yes or no), hepatic decompensation (presence of hepatic encephalopathy, variceal bleed, or ascites), alcohol use (never, prior, or current), and MELD-Na score at referral.

Our primary outcome was receipt of LT referral (referred vs not referred), defined as a clinician statement regarding referral for LT in the clinical documentation with additional supporting documentation such as faxing of patient coversheet to a transplant center. Secondary outcomes included completion of LT evaluation and wait-listing for LT. We defined evaluation for LT as successful completion of an initial appointment with a transplant hepatologist at the transplant center that received the referral. Reasons for nonreferral and non–wait-listing were obtained from clinical documentation from SNH hepatology clinic visits and transplant center evaluation visits, respectively.

### Statistical Analysis

We used frequencies and percentages to report categorical data. Medians and first to third quartiles (IQR) were used to summarize continuous variables. We compared variables between groups using Pearson χ^2^ and Wilcoxon rank sum tests for categorical and continuous variables, respectively.

We assessed factors associated with referral outcome using univariate logistic regression. Collinearity was assessed using a Pearson correlation matrix (coefficients were all <0.8). Using significant variables from our univariable analysis, we created 4 different multivariable logistic regression models assessing (1) association between patient demographics and the primary outcome, (2) association between socioeconomic proxies and the primary outcome, (3) association between disease-related variables and the primary outcome, and (4) association between all factors associated with the primary outcome from the first 3 models. All models were adjusted for hospital site (random effect).

We used multiple imputation to address missing data. Sample characteristics of participants were compared between those with or without missing data, where the missing data group was defined as those missing values for any variables. Given the significant differences between these 2 groups (eTable 1 in Supplement 1), we concluded that the data were not completely missing at random. Multiple imputation with 10 imputed data sets using a multiple chained equations approach was used to fill in missing values for race and ethnicity (4.8%), marital status (3.3%), documentation status (35.5%), housing status (21.3%), insurance type (3.5%), and alcohol use (3.3%). There were no notable differences in associations or estimates between the multivariable model results with unknown or missing categories and the multiple imputation multivariable model results. Results from the multiple imputation method were therefore presented as our study’s primary results.

Results are presented as odds ratios (ORs) and 95% CIs. Hypothesis tests were 2 sided, and the significance threshold was set to *P* < .05. Data were analyzed using Stata, version 17 (StataCorp LLC).

## Results

### Cohort Characteristics

The study cohort included 521 patients, with 298 (57.2%) from site 1, 142 (27.3%) from site 2, and 81 (15.5%) from site 3. Demographic and clinical characteristics by referral status are presented in Table 1. The median age was 60 years (IQR, 52-66) years; 156 patients (29.9%) were women and 365 (70.1%) were men. Hispanic or Latinx patients made up most of the cohort (311 [59.7%]), followed by non-Hispanic White (108 [20.7%]), non-Hispanic other (44 [8.4%]; includes American Indian or Alaska Native [n = 12], Asian American or Pacific Islander [n = 29], and >1 race or ethnicity [n = 4]), and non-Hispanic Black (33 [6.3%]) patients. Fourteen of the non-Hispanic Black patients (45.2%) were not referred on the basis of active alcohol use, and 2 (6.5%) were not referred due to medical contraindications. Two hundred forty-five patients (47.0%) were single, 287 (55.1%) were born outside the US, and 126 (24.2%) were undocumented. English was the most common language (262 [50.3%]), followed by Spanish (230 [44.1%]). Most of the cohort had stable housing (344 [66.0%]), while 66 (12.7%) had unstable housing. Most patients (338 [64.9%]) received Medicaid for insurance, while 74 (14.2%) had no insurance (Table 1).

**Table 1.** Patient Characteristics

Characteristic	Patient group^a^			*P* value^b^
	Total (N = 521)	Referred (n = 145)	Not referred (n = 376)	
Site				
1	298 (57.2)	99 (68.3)	199 (52.9)	<.001
2	142 (27.3)	22 (15.2)	120 (31.9)	
3	81 (15.5)	24 (16.6)	57 (15.2)	
Patient variables				
Age, median (IQR), y	60 (52-66)	62 (55-66)	59 (52-66)	.19
Sex				
Men	365 (70.1)	85 (58.6)	280 (74.5)	<.001
Women	156 (29.9)	60 (41.4)	96 (25.5)	
Race and ethnicity				
Hispanic or Latinx	311 (59.7)	99 (68.3)	212 (56.4)	<.001
Non-Hispanic Black	33 (6.3)	2 (1.4)	31 (8.2)	
Non-Hispanic White	108 (20.7)	20 (13.8)	88 (23.4)	
Non-Hispanic other^c^	44 (8.4)	12 (8.3)	32 (8.5)	
Unknown or missing	25 (4.8)	12 (8.3)	13 (3.5)	
Marital status				
Single	245 (47.0)	61 (42.1)	184 (48.9)	.005
Married	157 (30.1)	60 (41.4)	97 (25.8)	
Divorced, separated, or widowed	102 (19.6)	21 (14.5)	81 (21.5)	
Unknown or missing	17 (3.3)	3 (2.1)	14 (3.7)	
Born outside the US	287 (55.1)	95 (65.5)	192 (51.1)	.003
Documentation status				
Documented^d^	210 (40.3)	79 (54.5)	131 (34.8)	<.001
Undocumented	126 (24.2)	30 (20.7)	96 (25.5)	
Unknown or missing	185 (35.5)	36 (24.8)	149 (39.6)	
Primary language				
English	262 (50.3)	65 (44.8)	197 (52.4)	.27
Spanish	230 (44.1)	70 (48.3)	160 (42.6)	
Other^e^	29 (5.6)	10 (6.9)	19 (5.1)	
Socioeconomic status variables				
Housing				
Unstable^f^	66 (12.7)	10 (6.9)	56 (14.9)	.05
Stable	344 (66.0)	103 (71.0)	241 (64.1)	
Unknown or missing	111 (21.3)	32 (22.1)	79 (21.0)	
Insurance type				
Medicaid	338 (64.9)	107 (73.8)	231 (61.4)	.009
Medicare	82 (15.7)	23 (15.9)	59 (15.7)	
Uninsured	74 (14.2)	9 (6.2)	65 (17.3)	
Private	9 (1.7)	3 (2.1)	6 (1.6)	
Unknown or missing	18 (3.5)	3 (2.1)	15 (4.0)	
Liver disease variables				
Diabetes	129 (24.8)	49 (33.8)	80 (21.3)	.003
Hypertension	123 (23.6)	36 (24.8)	87 (23.1)	.68
Decompensation	423 (81.2)	121 (83.4)	302 (80.3)	.41
Ascites	379 (72.7)	108 (74.5)	271 (72.1)	.58
Hepatic encephalopathy	183 (35.1)	56 (38.6)	127 (33.8)	.30
Variceal bleed	80 (15.4)	31 (21.4)	49 (13.0)	.02
Alcohol use				
Current	127 (24.4)	18 (12.4)	109 (29.0)	<.001
Prior	300 (57.6)	93 (64.1)	207 (55.1)	
Never	77 (14.8)	32 (22.1)	45 (12.0)	
Unknown or missing	17 (3.3)	2 (1.4)	15 (4.0)	
Etiology				
NASH or NAFLD	43 (8.3)	22 (15.2)	21 (5.6)	.002
ALD	280 (53.7)	69 (47.6)	211 (56.1)	
Hepatitis B virus	17 (3.3)	7 (4.8)	10 (2.7)	
Hepatitis C virus	141 (27.1)	33 (22.8)	108 (28.7)	
Other^g^	40 (7.7)	14 (9.7)	26 (6.9)	
MELD-Na score at baseline				
15-20	338 (64.9)	95 (65.5)	243 (64.6)	.95
21-25	118 (22.6)	33 (22.8)	85 (22.6)	
>25	65 (12.5)	17 (11.7)	48 (12.8)	

Abbreviations: ALD, alcohol-associated liver disease; MELD-Na, model for end-stage liver disease–sodium; NAFLD, nonalcoholic fatty liver disease; NASH, nonalcoholic steatohepatitis.

^a^ Unless otherwise indicated, data are expressed as No. (%) of patients. Percentages have been rounded and may not total 100.

^b^ Calculated from Pearson χ^2^ and Wilcoxon rank sum tests for categorical and continuous variables, respectively.

^c^ Includes American Indian or Alaska Native (n = 12), Asian American or Pacific Islander (n = 29), and more than 1 race (n = 3).

^d^ Includes permanent residents, green card or visa holders, and citizens.

^e^ Other language includes Tigrinya, Punjabi, Arabic, Samoan, Chinese, Korean, Khmer, Thai, Tagalog, Russian, and Vietnamese.

^f^ Includes single room occupation or homeless.

^g^ Includes primary biliary cholangitis, primary sclerosing cholangitis, and cryptogenic and autoimmune hepatitis.

Of disease-related characteristics, 129 (24.8%) had diabetes and 123 (23.6%) had hypertension. Most of the cohort had a history of liver decompensation (423 [81.2%]), the most common being ascites (379 [72.7%]), followed by hepatic encephalopathy (183 [35.1%]) and variceal bleed (80 [15.4%]). The most common liver disease etiology was ALD (280 [53.7%]), followed by hepatitis C virus infection (141 [27.1%]) and nonalcoholic steatohepatitis or nonalcoholic fatty liver disease (43 [8.3%]). Most patients reported prior alcohol use (300 [57.6%]), followed by current use (127 [24.4%]) and no alcohol use (77 [14.8%]). At the index visit, MELD-Na scores were 15 to 20 among 338 patients (64.9%), 21 to 25 among 118 (22.6%), and greater than 25 among 65 (12.5%), with a median score of 19 (IQR, 16-22).

### SNH LT Postreferral Outcome

Of 521 patients, only 145 (27.8%) were referred for LT over a median follow-up of 30 (IQR, 5-65) months. Referral rates varied by site (99 of 298 [33.2%] at site 1, 22 of 142 [15.5%] at site 2, and 24 of 81 [29.6%] at site 3). Of the 376 patients not referred, active alcohol use or limited sobriety (123 [32.7%]) and insurance issues (80 [21.3%]) were the most common reasons for no referral, while clinical improvement or too early in the disease (60 [16.0%]), medical comorbidity (32 [8.5%]), lack of social support (15 [4.0%]), unknown reasons (16 [4.3%]), patient preference (12 [3.2%]), reassessment (9 [2.4%]), undocumented status (7 [1.9%]), substance reuse (7 [1.9%]), unstable housing (6 [1.6%]), nonadherence (4 [1.1%]), psychiatric illness (4 [1.1%]), and incarceration (1 [0.3%]) were the remaining reasons for no referral.

Of the 145 patients referred for LT, 106 (73.1%) were evaluated, 51 (35.2%) were wait-listed, and, ultimately, 28 (19.3%) received an LT (Figure). Variation in postreferral outcomes were observed by SNH site (eTable 2 in Supplement 1). Fifty-four patients were denied a place on the wait list; the main reasons for wait-list denial included insurance issues (18 [33.3%]), substance reuse (8 [14.8%]), psychiatric illness (8 [14.8%]), medical comorbidity (8 [14.8%]), death (6 [11.1%]) and lack of social support (3 [5.6%]). Of the 51 wait-listed patients, 10 (19.6%) were removed from the wait list and 3 (5.9%) had an unknown outcome. Of the 38 patients who remained on the wait list, 10 (26.3%) did not receive an LT, with the main reason being that they were too sick (3 [33.3%]). At the end of the study period (by May 1, 2022), 4 patients (19.0%) were still on the wait list and 7 (33.3%) had died. Excluding patients with clear psychosocial contraindications for LT (n = 282), including inadequate insurance, unstable housing, lack of social support, and active alcohol consumption or limited alcohol sobriety, the rate of referral increased to 145 (51.4%), with 106 (37.6%) evaluated, 51 (18.1%) wait-listed, and 28 (9.9%) receiving an LT (eFigure in Supplement 1). Clinical improvement and medical comorbidity were the 2 top reasons for nonreferral in this group.

**Figure.**
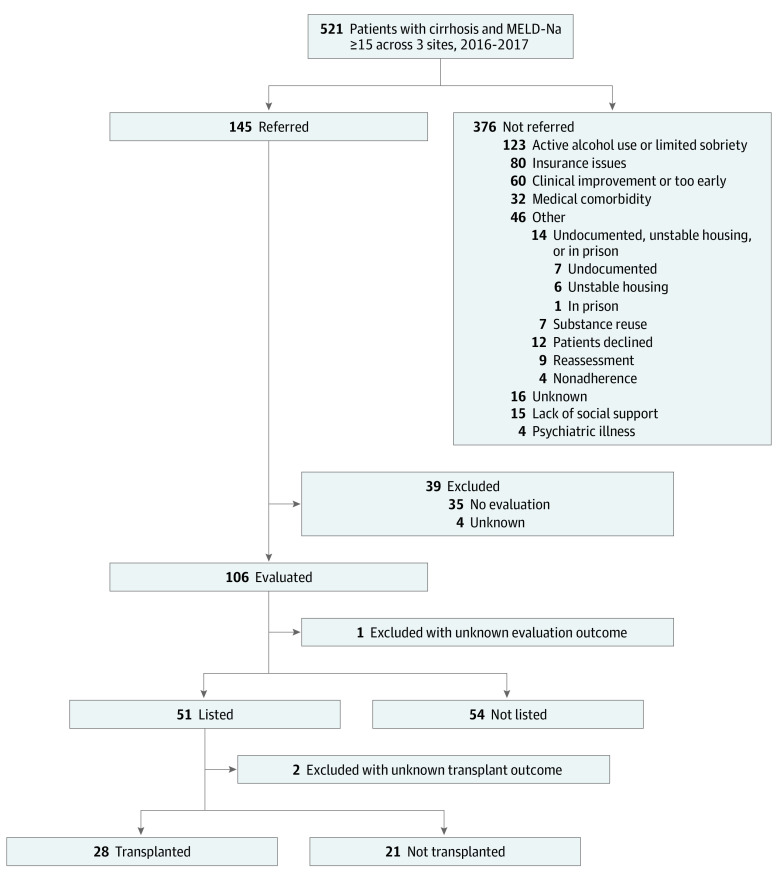
Burden of Liver Disease and Referral Outcome in the Population Treated in Safety-Net Hospitals Percentile calculation is based on the whole cohort (N = 521). MELD-Na indicates model for end-stage liver disease–sodium.

### Factors Associated With Referral Outcome

On univariate analysis, variables for LT referral were male sex, race and ethnicity, marital status, birth status outside the US, documentation status, insurance type, diabetes, history of variceal bleed, alcohol use, liver disease etiology, and hospital (eTable 2 in Supplement 1). Multivariable logistic regression was first performed for patient demographic variables (Table 2, model 1). In this model, male patients were 58% less likely to be referred (adjusted OR [AOR], 0.42 [95% CI, 0.27-0.66]). Compared with Hispanic or Latinx patients, non-Hispanic Black patients had 81% lower odds of being referred (AOR, 0.19 [95% CI, 0.04-0.83]). Patients who were married were 75% more likely to be referred compared with single patients (AOR, 1.75 [95% CI, 1.08-2.83]). While patients who were undocumented were 69% less likely to be referred than those who were documented (AOR, 0.31 [95% CI, 0.17-0.57]), patients who were born outside the US (AOR, 1.89 [95% CI, 1.03-3.48]) were 89% more likely to be referred than US-born patients. The second multiple imputation multivariable model assessed variables that could serve as proxies for socioeconomic status, including insurance type and housing status (Table 2, model 2). Patients who had unstable housing were 74% less likely to get referred (AOR, 0.36 [95% CI, 0.18-0.73]); those with no insurance were 59% less likely to get referred than patients with Medicaid (AOR, 0.41 [95% CI, 0.19-0.88]). The third model included disease-related variables (Table 2, model 3). In this model, patients with current alcohol use were 69% less likely to be referred than patients with no history of alcohol use (AOR, 0.31 [95% CI, 0.14-0.69]).

**Table 2.** Multiple Imputation Multivariable Logistic Regression Models for Referral Outcome

Variable	AOR (95% CI)			
	Model 1 (demographic characteristics)	Model 2 (socioeconomic characteristics)	Model 3 (disease variables)	Model 4 (significant factors from models 1-3)
Site				
1	1 [Reference]	1 [Reference]	1 [Reference]	1 [Reference]
2	0.34 (0.17-0.72)^a^	0.38 (0.21-0.69)^a^	0.36 (0.20-0.62)^a^	0.40 (0.18-0.87)^a^
3	1.11 (0.58-2.10)	0.90 (0.52-1.57)	0.83 (0.47-1.48)	1.15 (0.59-2.22)
Male sex	0.42 (0.27-0.66)^a^	NA	NA	0.50 (0.31-0.81)^a^
Race and ethnicity				
Hispanic or Latinx	1 [Reference]	1 [Reference]	1 [Reference]	1 [Reference]
Non-Hispanic Black	0.19 (0.04-0.83)^a^	NA	NA	0.19 (0.04-0.89)^a^
Non-Hispanic White	0.87 (0.41-1.84)	NA	NA	0.77 (0.36-1.68)
Non-Hispanic other^b^	0.72 (0.32-1.64)	NA	NA	0.61 (0.26-1.46)
Marital status				
Single	1 [Reference]	1 [Reference]	1 [Reference]	1 [Reference]
Married	1.75 (1.08-2.83)^a^	NA	NA	1.53 (0.94-2.51)
Divorced, separated, or widowed	0.84 (0.46-1.53)	NA	NA	0.81 (0.43-1.50)
Documentation status				
Documented^c^	1 [Reference]	1 [Reference]	1 [Reference]	1 [Reference]
Undocumented	0.31 (0.17-0.57)^a^	NA	NA	0.34 (0.19-0.62)^a^
Born outside the US	1.89 (1.03-3.48)^a^	NA	NA	1.59 (0.84-3.02)
Housing status				
Stable	1 [Reference]	1 [Reference]	1 [Reference]	1 [Reference]
Unstable^d^	NA	0.36 (0.18-0.73)^a^	NA	0.51 (0.24-1.08)
Insurance type				
Medicaid	1 [Reference]	1 [Reference]	1 [Reference]	1 [Reference]
Private	NA	1.87 (0.42-8.34)	NA	1.41 (0.28-7.09)
Medicare	NA	1.11 (0.62-2.01)	NA	0.94 (0.49-1.80)
Uninsured	NA	0.41 (0.19-0.88)^a^	NA	0.40 (0.18-0.89)^a^
Diabetes	NA	NA	1.46 (0.89-2.39)	NA
Variceal bleed	NA	NA	1.71 (1.01-2.89)	NA
Alcohol use				
Never	1 [Reference]	1 [Reference]	1 [Reference]	1 [Reference]
Current	NA	NA	0.31 (0.14-0.69)^a^	0.38 (0.17-0.86)^a^
Prior	NA	NA	0.75 (0.40-1.41)	0.72 (0.37-1.38)
Etiology				
Hepatitis C virus	1 [Reference]	1 [Reference]	1 [Reference]	1 [Reference]
NASH or NAFLD	NA	NA	1.71 (0.76-3.86)	NA
Hepatitis B virus	NA	NA	1.63 (0.54-4.99)	NA
ALD	NA	NA	0.91 (0.53-1.58)	NA
Other^e^	NA	NA	1.26 (0.56-2.86)	NA

Abbreviations: ALD, alcohol-associated liver disease; AOR, adjusted odds ratio; NA, not applicable; NAFLD, nonalcoholic fatty liver disease; NASH, nonalcoholic steatohepatitis.

^a^ *P* < .05.

^b^ Includes American Indian or Alaska Native, Asian American or Pacific Islander, and more than 1 race or ethnicity.

^c^ Includes permanent residents, green card or visa holders, and citizens.

^d^ Includes single room occupation or homeless.

^e^ Includes primary biliary cholangitis, primary sclerosing cholangitis, and cryptogenic and autoimmune hepatitis.

Our final model included all significant factors associated with LT referral from models 1 through 3 (Table 2, model 4). In this model, male sex (AOR, 0.50 [95% CI, 0.31-0.81]), non-Hispanic Black race (AOR, 0.19 [95% CI, 0.04-0.89]), undocumented status (AOR, 0.34 [95% CI, 0.19-0.62]), no insurance (AOR, 0.40 [95% CI, 0.18-0.89]), and current alcohol use (AOR, 0.38 [95% CI, 0.17-0.86]) were associated with lower odds of being referred for LT evaluation. All models were adjusted for site, with site 2 being less likely to refer patients when compared with site 1 (Table 2).

## Discussion

In this multicenter SNH study, only 1 in 3 patients with ESLD and at least 1 indication for LT (MELD-Na score ≥15) were referred, 1 in 10 were wait-listed, and only 28 (5.4%) received an LT over 3 years of follow-up. A study conducted before the MELD-Na era^16^ found that only 3% of patients discharged with a liver-related condition underwent LT evaluation. In a study of patients with a MELD-Na score of 15 or greater in the Veterans Affairs health care system,^17^ only 4.5% were referred, 3.0% were wait-listed, and 1.6% underwent LT. Taken together, referral is clearly an early barrier to LT. Our findings are 4-fold: (1) approximately one-quarter of patients were not referred due to sociodemographic barriers (eg, insurance, documentation status, housing stability); (2) more than one-quarter of patients were not referred due to active alcohol use or limited sobriety; (3) non-Hispanic Black patients had lower odds of referral; and (4) there were variations in referral practices by hospital site. Our study highlights the challenges that patients with cirrhosis in SNHs face in accessing a life-saving resource and the importance of dedicated investigation and targeted interventions into this population and its primary source of health care.

Most patients in SNHs (>70%) were not referred, with social determinants of health (including uninsured status, undocumented status, and unstable housing) all independently associated with lower odds of being referred. These findings demonstrate the frequency with which patients with ESLD and low socioeconomic status experience structural barriers,^3^ resulting in a minority of LT recipients (8%) arising from SNH referrals.^9^ Not surprisingly, uninsured patients were 60% less likely to get referred when compared with those with Medicaid, concordant with previous studies on LT disparities related to insurance.^17,18,19,20^ Policies to expand insurance access and simplify access to coverage—such as Medicare Advantage coverage for patients with end-stage kidney disease or state-level Medicaid expansion—might help address insurance-related inequities. Currently, Hospital Presumptive Eligibility allows states to enroll hospitalized low-income patients in temporary Medicaid coverage^21,22^; a similar model could be instated for outpatients with ESLD, allowing enrollment in temporary Medicaid coverage with a plan to transition to sustained coverage. As all of our SNH sites were in Medicaid expansion states with robust Medicaid programs, insurance status as a barrier to LT referral is likely further compounded in other parts of the country.

Studying and accurately capturing data on undocumented or nonlegal US residents is challenging, but they remain an important group to characterize.^23^ In this study, undocumented patients had 66% to 69% lower odds of LT referral. In UNOS data, only 0.4% of LT recipients were nonresident and non-US citizens or residents (59% Hispanic and 51% with Medicaid).^24^ While constituting a small percentage of the LT wait list, they predominantly seek care in SNHs, and the process of obtaining emergency insurance and concerns regarding deportation are additional barriers experienced. In many states, undocumented patients are ineligible for insurance and therefore have no access to LT. Effective May 2022 in California, the process of obtaining full-scope medical insurance was simplified such that any patient older than 50 years is automatically eligible regardless of immigration status^25^; such policies will hopefully streamline referrals and expand access to LT for this important underserved population.

Alcoholic liver disease was the most common etiology of ESLD among patients in SNHs: 53.7% of our cohort had ALD, 24.4% had current alcohol use, and 32.7% of patients not referred for LT had active alcohol use or limited sobriety. Current alcohol use was associated with 62% to 69% lower odds of being referred when compared with no prior alcohol use, although ALD itself was not associated with referral. Over the time period of our study, LT for ALD had largely been guided by the 6-month rule that mandates at least 6 months of abstinence before LT consideration.^26,27,28^ During the study period, all 3 sites were guided by the 6-month rule. Early LT for ALD, defined as less than 6 months of pre-LT abstinence, has been increasingly adopted—although variably so across transplant centers—and favors White, male, and privately insured patients.^29,30^ Given the high prevalence of ALD among patients in SNHs, access to alcohol cessation resources and addiction specialists in this clinical setting are paramount. Similarly, a multidisciplinary approach at transplant centers is needed, as addiction medicine was available at only 2 of the transplant centers in our study (neither embedded within transplant clinics), while 2 centers had access to transplant psychiatry services only. Importantly, educating SNH staff on the present availability of early LT for ALD at more than 70% of transplant centers,^31^ including the factors that might be associated with a successful referral as well as implementing more standardized policies around limited sobriety, are important steps to prevent further disparities in access to LT for underinsured, low-income, and racial and ethnic minority patients with ALD.

Non-Hispanic Black patients in our cohort were 81% less likely to be referred for LT when compared with Hispanic or Latinx patients, the largest SNH racial or ethnic group among our sites. Studies have consistently found lower rates of wait-listing, more advanced disease at wait-listing, and higher waiting list–related mortality among Black patients.^16,32,33,34^ Although transplant rates for Black patients improved after MELD-Na implementation, this population continues to be underrepresented on the wait list.^35^ In a Veterans Affairs study,^32^ Black patients were less likely to have LT referral mentioned in their medical encounters. While our findings must be taken in the context of the small number of Black patients (6.3%), they suggest that barriers to LT access for Black patients start at the referral stage. Fourteen Black patients in our SNH study (45.2%) were not referred on the basis of active alcohol use, and only 2 (6.5%) were not referred due to medical contraindications, emphasizing the critical need for substance use support in SNH settings.

Referral rates varied by site (15.5%-33.2%). While all SNH sites were in urban settings, referral practice variations likely exist. While there were no standard criteria for referral at any site, sites varied in whether clinicians were transplant hepatology trained or even directly affiliated with a transplant center, which might influence the likelihood of referral. In addition, referral patterns might depend on local transplant center wait-listing and LT practices, especially regarding insurance, citizenship, and alcohol-related policies. Further in-depth studies are needed to understand the impact of clinician- and system-level practice variations on referral and to develop uniform practice guidelines.

### Limitations

This study has limitations, given its retrospective study design, although we were able to obtain comprehensive data for referral denial that otherwise would not be available from large databases. Although our study cohort represents 3 different SNH sites across 2 different UNOS regions on the West Coast with a diverse patient population, there might be selection bias, and our results may not be generalizable to safety-net settings in other geographic regions. Second, given the lack of standardized local protocols in referral and wait-listing practices, there were variations in the site-specific proportion of patients who progressed through the LT process. Third, we did not include patients initially seen and referred in the inpatient setting. By limiting our study population to patients seen in outpatient hepatology clinics, we sought to limit heterogeneity and the influence of external factors, such as local protocols in hospital transfers and bed availability. Fourth, we had missing data for certain demographic variables, which differed by referral outcome, precluding complete case analysis; thus, we used multiple imputation to impute missing variables. Finally, our study population did not include those with other important indications for LT such as hepatocellular carcinoma among those with compensated cirrhosis.

## Conclusions

In this cohort study, nearly three-fourths of patients with ESLD in SNHs were not referred for LT evaluation. Social determinants of health and ALD played a large role in nonreferral. In SNH settings that serve the most vulnerable patients, improving LT referral will require a multipronged approach at the patient, clinician, and system levels.

## Notes

Supplement 1. **eTable 1.** Sample Characteristics of Participants Missing Data vs Those Not Missing Data **eTable 2.** Breakdown of Referral, Evaluation, Waitlist, and Transplant Rates by Safety Net Hospital **eTable 3.** Univariate Logistic Regression Models for Referral Outcome **eFigure.** Referral Outcomes in Patients With No Psychosocial Contraindications for LT Evaluation

Supplement 2.
Data Sharing Statement
