# Supplementary material for: Factors Associated With Liver Transplant Referral Among Patients With Cirrhosis at Multiple Safety-Net Hospitals
Source: JAMA Netw Open. 2023 Jun 8;6(6):e2317549. doi: 10.1001/jamanetworkopen.2023.17549 (PMC10251211; doi:10.1001/jamanetworkopen.2023.17549)
Supplement: Supplement 1. — eTable 1. Sample Characteristics of Participants Missing Data vs Those Not Missing Data eTable 2. Breakdown of Referral, Evaluation, Waitlist, and Transplant Rates by Safety Net Hospital eTable 3. Univariate Logistic Regression Models for Referral Outcome eFigure. Referral Outcomes in Patients With No Psychosocial Contraindications for LT Evaluation [file jamanetwopen-e2317549-s001.pdf]

Supplementary Online Content

Yilma M, Kim NJ, Shui AM, et al. Factors associated with liver transplant referral among patients with cirrhosis at multiple safety-net hospitals. *JAMA Netw Open*. 2023;6(6):e2317549. doi:10.1001/jamanetworkopen.2023.17549

eTable 1. Sample Characteristics of Participants Missing Data vs Those Not Missing Data
eTable 2. Breakdown of Referral, Evaluation, Waitlist, and Transplant Rates by Safety Net Hospital
eTable 3. Univariate Logistic Regression Models for Referral Outcome
eFigure. Referral Outcomes in Patients With No Psychosocial Contraindications for LT Evaluation

This supplementary material has been provided by the authors to give readers additional information about their work.

© 2023 Yilma M et al. *JAMA Network Open*.

eTable 1. Sample Characteristics of Participants Missing Data vs Those Not Missing Data

Variable	Cases Not Missing Data N = 220	Cases with Missing Data N = 301	p-value
			<.001
Site 1	147 (66.8)	151 (50.2)	
Site 2	2 (0.9)	140 (46.5)	
Site 3	71 (32.3)	10 (3.3)	
**Sex**			0.87
Male	155 (70.5)	210 (69.8)	
Female	65 (29.6)	91 (30.2)	
**Race and Ethnicity**			<.001
Non-Hispanic White patients	26 (11.8)	82 (27.2)	
Non-Hispanic Black patients	16 (7.3)	17 (5.7)	
Hispanic/Latinx patients	165 (75.0)	146 (48.5)	
Non-Hispanic Other[a]	13 (5.9)	31 (10.3)	
Unknown/missing	0 (0.0)	25 (0.3)	
**Marital Status**			0.003
Single	112 (50.9)	133 (44.2)	
Married	67 (30.5)	90 (29.9)	
Divorced/Separated/Widowed	41 (18.6)	61 (20.3)	
Unknown/Missing	0 (0.0)	17 (5.7)	
**Foreign Born**	141 (64.1)	146 (48.5)	<.001
**Documentation Status**			<.001
Not Undocumented	136 (61.8)	74 (24.6)	
Undocumented	84 (38.2)	42 (14.0)	
Unknown/missing	0 (0.0)	185 (61.5)	
**Housing**			<.001
Unstable Housing[b]	36 (12.7)	30 (10.0)	
Stable Housing	184 (83.6)	160 (53.2)	
Unknown/missing	0 (0.0)	111 (36.9)	
**Insurance type**			<.001
Medicaid	179 (81.4)	159 (52.8)	
Medicare	23 (10.5)	59 (19.6)	
Uninsured	18 (8.2)	56 (18.6)	
Private	0 (0.0)	9 (3.0)	
Unknown/missing	0 (0.0)	18 (6.0)	
**Diabetes**	53 (24.1))	76 (25.3)	0.76
**Variceal bleed**	42 (19.1)	38 (12.6)	0.04
**Alcohol use**			0.001
Current	50 (22.7)	77 (25.6)	
Prior	141 (64.1)	159 (52.8)	
Never	29 (13.2)	48 (16.0)	
Unknown/missing	0 (0.0)	17 (5.7)	
**Etiology**			0.13
NASH/NAFLD	18 (8.2)	25 (8.3)	
Alcohol-associated liver disease (ALD)	129 (58.6)	151 (50.2)	
Hepatitis B Virus (HBV)	3 (1.4)	14 (4.7)	
Hepatitis C Virus (HCV)	53 (24.1)	88 (29.2)	
Other[c]	17 (7.7)	23 (7.6)	

Missing Data Cases represent participants missing values for any variables included in the multivariable models

[a]other race and ethnicity includes Asian American/Pacific Islander patients (n=29), American Indian/Alaskan Native patients (n=12), Multiracial (n=4)

[b]Unstable Housing = Single Room Occupation, homeless

[c]other etiology: Primary Biliary cholangitis (PBC), Primary sclerosing cholangitis (PSC), Cryptogenic and Autoimmune hepatitis

p-values are from Pearson chi-squared and Wilcoxon rank sum tests for categorical and continuous variables, respectively

© 2023 Yilma M et al. *JAMA Network Open*.

eTable 2. Breakdown of Referral, Evaluation, Waitlist, and Transplant Rates by Safety Net Hospital

Variable	Total (%)	Referred (%)	Waitlisted (%)	Transplanted (%)
Site 1	298	99 (33.2)	38 (38.4)	24 (63.2)
Site 2	142	22 (15.5)	5 (22.7)	2 (40.0)
Site 3	81	24 (29.6)	8 (33.3)	2 (25.0)
Total	521	145 (27.8)	51 (35.2)	28 (54.9)

© 2023 Yilma M et al. *JAMA Network Open*.

eTable 3. Univariate Logistic Regression Models for Referral Outcome

Variable	OR (95% CI)	p-value
**Age (years)**	1.01 (0.99, 1.03)	0.328
**Male sex**	0.49 (0.32, 0.73)	<.001
**Race/Ethnicity (***ref = Hispanic/Latinx***)**		<.001
White	0.49 (0.28, 0.84)	0.009
Black	0.14 (0.03, 0.59)	0.007
Other[a]	0.80 (0.40, 1.63)	0.54
Unknown/missing	1.98 (0.87, 4.49)	0.10
**Marital Status** (*ref = single*)		0.006
Married	1.87 (1.21, 2.88)	0.005
Divorced/Widowed/Separated	0.78 (0.45, 1.37)	0.39
Unknown/Missing	0.65 (0.18, 2.33)	0.50
**Foreign Born**	1.82 (1.22, 2.71)	0.003
**Documentation Status** *(ref = not undocumented)*		<.001
Undocumented	0.52 (0.32, 0.85)	0.009
Unknown/missing	0.40 (0.25, 0.63)	<.001
**Primary language (***ref = English***)**		
Spanish	1.33 (0.90, 1.98)	0.16
Other[b]	1.52 (0.67, 3.40)	0.31
**Housing Status** *(ref = stable housing)*		0.05
Unstable Housing[c]	0.42 (0.21, 0.85)	0.02
Unknown/missing	0.95 (0.59, 1.52)	0.82
**Insurance type (***ref = Medicaid)*		<.001
Private	1.08 (0.26, 4.40)	0.92
Medicare	0.84 (0.49, 1.43)	0.53
Uninsured	0.30 (0.14, 0.62)	0.001
Unknown/missing	0.43 (0.12, 1.52)	0.19
**Diabetes**	1.90 (1.24, 2.88)	0.003
**Hypertension**	1.10 (0.70, 1.71)	0.68
**Hepatic Decompensation (Ascites, HE, Variceal bleed)**	1.24 (0.74, 2.05)	0.41
**Ascites**	1.13 (0.73, 1.75)	0.58
**Hepatic Encephalopathy**	1.23 (0.83, 1.83)	0.30
**Variceal Bleed**	1.81 (1.10, 3.00)	0.02
**Alcohol use** *(ref = never/prior)*		<.001
Current	0.33 (0.19, 0.57)	<.001
Unknown/missing	0.27 (0.06, 1.19)	0.08
**Disease Etiolog***y (ref = HCV)*		0.003
NASH/NAFLD	3.42 (1.70, 7.00)	0.001
HBV	2.29 (0.81, 6.49)	0.12
Alcoholic liver disease (ALD)	1.07 (0.66, 1.72)	0.78
Other[d]	1.76 (0.83, 3.76)	0.14
**MELD at baseline** *(ref = 15 – 20)*		
21 – 25	0.99 (0.62, 1.58)	0.98
> 25	0.91 (0.50, 1.65)	0.75
**Site** *(ref = Site 1)*		<.001
Site 2	0.37 (0.22, 0.62)	<.001
Site 3	0.85 (0.50, 1.44)	0.54

© 2023 Yilma M et al. *JAMA Network Open*.

[a]other race and ethnicity includes Asian American/Pacific Islander patients (n=29), American Indian/Alaskan Native patients (n=12), Multiracial (n=4)
[b]Other language includes Tigrinya, Punjabi, Arabic, Samoan, Chinese, Korean, Khmer, Thai, Tagalog, Russian, and Vietnamese
[c]Unstable Housing = Single Room Occupation, homeless
[d]other etiology: Primary Biliary cholangitis (PBC), Primary sclerosing cholangitis (PSC), Cryptogenic and Autoimmune hepatitis

© 2023 Yilma M et al. *JAMA Network Open*.

eFigure. Referral Outcomes in Patients With No Psychosocial Contraindications for LT Evaluation

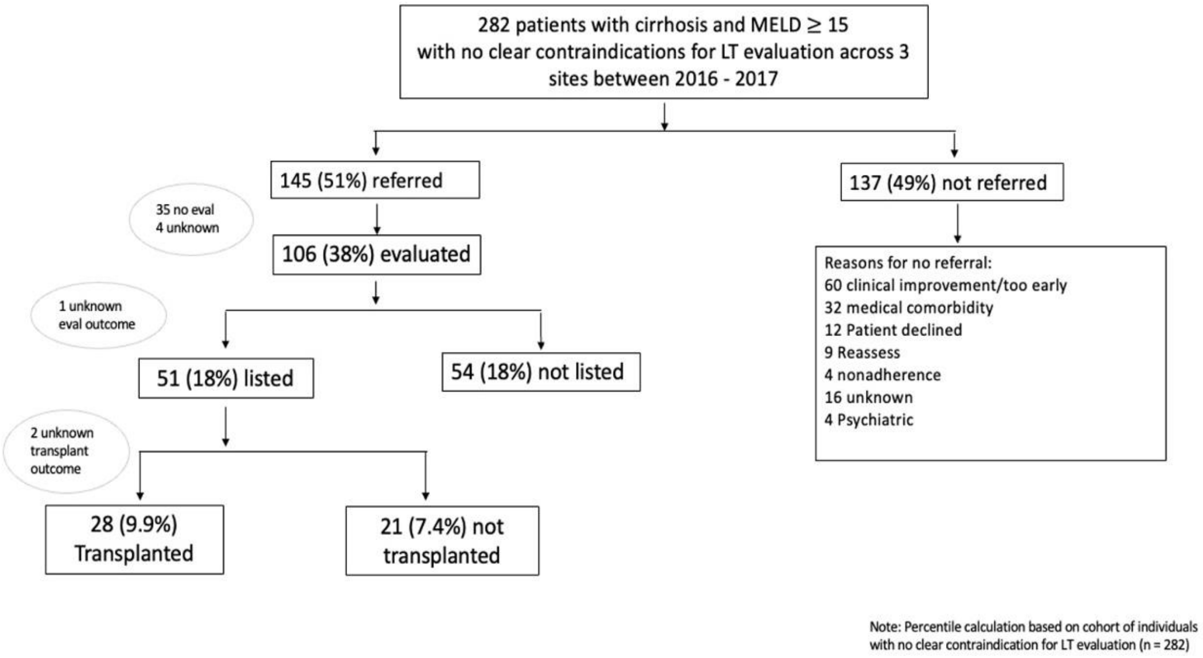

© 2023 Yilma M et al. *JAMA Network Open*.
