# Supplementary material for: Factors Associated With Liver Transplant Referral Among Patients With Cirrhosis at Multiple Safety-Net Hospitals
Source: JAMA Netw Open. 2023 Jun 8;6(6):e2317549. doi: 10.1001/jamanetworkopen.2023.17549 (PMC10251211; doi:10.1001/jamanetworkopen.2023.17549)
Supplement: Supplement 2. — Data Sharing Statement [file jamanetwopen-e2317549-s002.pdf]

Data Sharing Statement

Yilma. Factors Associated With Liver Transplant Referral Among Patients With Cirrhosis at Multiple Safety-Net Hospitals. *JAMA Netw Open*. Published June 08, 2023. doi:10.1001/jamanetworkopen.2023.17549

Data
Data available: No
